# Rheological Characterization of Isabgol Husk, Gum Katira Hydrocolloids, and Their Blends

**DOI:** 10.1155/2014/506591

**Published:** 2014-08-25

**Authors:** Vipin Kumar Sharma, Bhaskar Mazumder, Vinod Nautiyal

**Affiliations:** ^1^Department of Pharmaceutical Sciences, Faculty of Medical Science & Health, Gurukul Kangri University, Haridwar, Uttarakhand 249404, India; ^2^Department of Pharmaceutical Sciences, Dibrugarh University, Dibrugarh, Assam 786004, India

## Abstract

The rheological parameters of Isabgol husk, gum katira, and their blends were determined in different media such as distilled water, 0.1 N HCl, and phosphate buffer (pH 7.4). The blend properties of Isabgol husk and gum katira were measured for four different percentage compositions in order to understand their compatibility in dispersion form such as 00 : 100, 25 : 50, 50 : 50, 75 : 25, and 100 : 00 in the gel strength of 1 mass%. The miscibility of blends was determined by calculating Isabgol husk-gum katira interaction parameters by Krigbaum and Wall equation. Other rheological properties were analyzed by Bingham, Power, Casson, Casson chocolate, and IPC paste analysis. The study revealed that the power flow index “*p*” was less than “1” in all concentrations of Isabgol husk, gum katira, and their blends dispersions indicating the shear-thinning (pseudoplastic) behavior. All blends followed pseudoplastic behavior at thermal conditions as 298.15, 313.15, and 333.15°K and in dispersion media such as distilled water, 0.1 N HCl, and phosphate buffer (pH 7.4). Moreover, the study indicated the applicability of these blends in the development of drug delivery systems and in industries, for example, ice-cream, paste, nutraceutical, and so forth.

## 1. Introduction

Gums and mucilages are widely used natural materials for food and pharmaceutical industries. The natural materials have advantages over synthetic ones since they are chemically inert, nontoxic, less expensive, biodegradable, and widely available [1]. These can also be modified in different ways to obtain tailor-made materials and thus can compete with the available synthetic polymers. The importance of biocompatible and biodegradable hydrophilic polymers has wide applications in different fields such as polymer engineering, chemical engineering, pharmaceuticals, food, and agriculture because of their propensity to combine with others [2–4]. The blends of these biopolymers are also of significant importance and recently have been investigated for application in drug delivery systems and in the field of foods science [5, 6]. In the plastic industry, polyvinyl alcohol blends with agar and hydroxyethylcellulose have been investigated in order to improve the mechanical properties of biodegradable films [5]. Such blends showed superior performances over the conventional individual polymers and, consequently, the range of applications have grown rapidly for such class of materials. In the recent years, carbohydrate and biodegradable polymers have been extensively used to develop the controlled release formulations of drugs having short plasma life. Amongst the various polymers employed, hydrophilic biopolymers are quite suitable because they are nontoxic and acceptable by the regulatory authorities [7]. The application of any natural gum or mucilage depends upon its viscosity. The choice of selecting the natural gum and its blends for sustained release effect depends upon its gelling strength [8]. The interacting blends of poly(acrylic acid) with poly(vinylpyrrolidone) or poly(vinyl alcohol) in aqueous solutions have been studied by ultrasonic, rheological, and viscometric techniques [9, 10]. Different types of interactions, such as electrostatic interaction, hydrogen bonding, and hydrophobic interaction, are established between biopolymers. In order to clarify the association of biopolymers through structure formation, it is important to study the molecular interactions of different kinds of biopolymers. It is thought that the structural formation of biopolymer blends is an important subject from both industrial and scientific points of view.

Isabgol husk is medicinally important polysaccharide and it has been reported for the treatment of constipation, diabetes, diarrhoea, inflammation bowl diseases, ulcerative colitis, cancer, obesity, high cholesterol, and so forth [11]. Husk mucilage obtained from the seed coat by mechanical milling/grinding of the outer layer of the seeds is fibrous and hydrophilic and forms the clear, colorless mucilaginous gel by absorbing water. Recently, the US Food and Drug Administration has authorized the use of food products containing soluble fiber from Isabgol husk [12]. A gastroretentive sustained release delivery system of ofloxacin has been developed with release polymers like psyllium husk and a swelling agent, crospovidone [13, 14].

Gum katira, an exudate from the bark of* Cochlospermum religiosum *(family Cochlospermaceae), is pale and semitransparent and insoluble in water but swells into a pasty transparent mass with water. It has obtained great importance in recent years and is exported annually from India for use in cigar, paste, and ice-cream industries [15]. The gum is sweet, thermogenic, anodyne, sedative, and useful in cough, diarrhea, dysentery, pharyngitis, gonorrhoea, syphilis, and trachoma [16]. It consists of equimolecular proportion of L-rhamnose, D-galactose, and D-galacturonic acid, together with traces of a ketohexose. It has been reported that [1→2]-4-linked galacturonic acid is present in the linear chain of this polysaccharide with similar residues of neutral sugars [17, 18].

This study has been carried out for rheological properties of blends comprising Isabgol husk and gum katira and is based on assessment of miscibility of Isabgol husk and gum katira blends in different concentrations. The effect of different thermal conditions was analyzed on blends miscibility and rheological characteristics.

## 2. Materials and Methods

Isabgol husk and gum katira were procured from local market. Other chemicals and regents such as potassium dihydrogen phosphate, sodium hydroxide, and hydrochloric acid of analytical grade were procured from Loba Chemie, Mumbai, and used as such without further purification and modification.

### 2.1. Preparation of Isabgol Husk and Gum Katira Dispersions

About 1 and 2 mass% dispersion of Isabgol husk and gum katira were prepared in distilled water and kept aside at room temperature for 6 h to remove the entrapped air and complete swelling. The blends of Isabgol husk and gum katira comprising each 1 mass% were prepared by thoroughly mixing the above dispersions in three percentage ratios such as 75 : 25, 50 : 50, and 25 : 75, respectively. The blends by applying 2 mass% of Isabgol husk and 1 mass% of gum katira dispersion and vice versa were also prepared in the above compositions for assessing the effect of polysaccharides concentrations on miscibility. Viscosities of these dispersions in pure and blend form were determined in triplicate (*n* = 3) by Brookfield's viscometer (Model: RV DV-E, USA) by taking 6.7 mL of the sample into a removable sample chamber. The removable sample chamber was inserted into the water jacket assembly and an insulation cap was placed on the chamber to maintain the temperature constant of dispersions samples during measurements. For rheological investigation, spindle-18 (SC-18) was selected to perform the study. The compatibility in terms of miscibility of Isabgol husk and gum katira in blends containing different strengths of these polysaccharides was analyzed at 298.15, 313.15, and 333.15°K.

### 2.2. Determination of Miscibility of Isabgol Husk-Gum Katira Blends

The miscibility of Isabgol husk and gum katira blends in dispersion form was studied by calculating the polymer-polymer interaction parameter “Δ*b*” of the blends using the Krigbaum and Wall equation [19]

(1) $$\begin{array}{c} b_{m}=x_{1}^{2}b_{11}+2x_{1}x_{2}b_{12}+x_{2}^{2}b_{22}, \end{array}$$

where “*x*_1_” and “*x*_2_” are the mass fraction of Isabgol husk and gum katira, respectively, “*b*_11_” and “*b*_22_” are the respective interaction parameters of Isabgol husk and gum katira dispersions, respectively, “*b*_12_” is the interaction parameter of the blend system, and “*b*_*m*_” represents the global interaction between two polymeric species, respectively. Here, the interaction parameters “*b*_11_”, “*b*_22_”, and “*b*_*m*_” were calculated from the slope of the plot of reduced viscosity of Isabgol husk and gum katira and their blends versus concentration, respectively [20]. The samples were studied in triplicate (*n* = 3) and the data were represented as the mean of the successive results with their respective standard deviations. The linear relationship found from such plots for the entire composition is generally the characteristic of blend compatibility.

The miscibility of these blends was also analyzed by calculating the reduced viscosity (*η*_sp_/*C* ^*^). Here, “*C* ^*^” is the concentration of individual polysaccharide in blends and alone. Then, from the nature of the plot of (*η*_sp_/*C* ^*^) versus *C* ^*^, blend compatibility was predicted. The values of intrinsic viscosity “[*η*]_*m*_” were calculated for both the individual polymers and their blends followed by extrapolation of “*η*_sp_/*C* ^*^” to zero concentration [21]. The values of the intrinsic viscosity “[*η*]_*m*_” obtained from such plots for the blends were also calculated theoretically by using the following expression, and compared

(2) $$\begin{array}{c} {\left[\eta \right]}_{m}={\left[\eta \right]}_{1}x_{1}+{\left[\eta \right]}_{2}x_{2}. \end{array}$$

The interaction parameter “*b*_12_^*^” was calculated theoretically by using the equation

(3) $$\begin{array}{c} b_{12}^{*}={\left(b_{1}b_{2}\right)}^{1/2}. \end{array}$$

Here, the value of “*b*_1_ *b*_2_” was the slope of the plots of reduced viscosity versus concentration of the individual polymers calculated by using classical Huggins equation [20]:

(4) $$\begin{array}{c} \frac{\eta_{\text{sp}}}{C^{*}}={\left[\eta \right]}_{0}+bC^{*}. \end{array}$$

Thus, the difference “Δ*b*” between the theoretically calculated “*b*_12_^*^” from the above equation and that of the experimental “*b*_12_” calculated from the equation was calculated as

(5) $$\begin{array}{c} \Delta b=\left(b_{12}-b_{12}^{*}\right). \end{array}$$

### 2.3. Determination of Rheological Behaviour of Blends by Rheological Models

The analysis of rheological data obtained through investigation was treated by following mathematical models [21]. Non-Newtonian behavior can be simply expressed through an equation and in some cases; the coefficients of a model can be used to draw the inference regarding the performance of a fluid under conditions of use. Newtonian flow is defined by a phenomenon of response in shear stress for a change in shear rate (linear relationship). Non-Newtonian fluids exhibit a nonlinear stress/rate relationship. The Newtonian equation for viscosity has been modified many times to characterize non-Newtonian behavior. Some of the widely used equations include the following:

(6) Casson  equation: τ=τ0+ηD,

(7) Casson  chocolate  equation: (1+a)τ=2τ0+(1+a)ηD,

(8) Bingham  plastic  equation:  τ=τ0+ηD,

(9) Power  law: τ=kDη,

(10) IPC  paste  analysis: η=kRs,

where “*τ*” is shear stress (N/m^2^), “*τ*_0_” yield stress/shear stress at zero shear rate (N/m^2^), “*D*” is shear rate (sec^−1^), “*a*” is spindle radius, “*K*” is consistency index (mPa·s), “*n*” is flow index, “*β*”, is consistency multiplier, “*R*” is rotational speed (sec^−1^), and “*s*” is shear sensitivity factor, respectively.

Using the magnitudes of “*K*” and “*n*”, apparent viscosity (*η*_app_) at shear rate of  1 sec^−1^ (60 rpm) was calculated. In addition, the effect of temperature (298.15, 313.15, and 333.15°K) on *η*_app,60 rpm_ was studied for Isabgol husk, gum katira, and their blends dispersions using the Arrhenius equation [21]:

(11) $$\begin{array}{c} \eta_{\text{app},60}=A\exp\left(\frac{E_{\text{act}}}{RT}\right), \end{array}$$

where *η*_app,60 rpm_ is the apparent viscosity (mPa*·*s) at 60 rpm (1 s^−1^), “*A*” is a constant (mPa*·*s), “*T*” is the absolute temperature (°K), “*R*” is the gas constant (8.3144 J/mol°K), and “*E*_act_” is the activation energy (kJ/mol), respectively.

The following equations were applied for the calculation of viscosity of blends from the individual viscosity data and from the volume of gum katira and Isabgol husk applied in the blends preparation. The rheological data obtained from individual polymeric dispersion of gum katira and Isabgol husk was compared with blends:

(12) $$\begin{array}{c} \eta =\eta_{1}\phi_{1}+\eta_{2}\phi_{2}, \end{array}$$

(13) $$\begin{array}{c} \eta_{\text{mix}}=\eta_{2}+\frac{\phi_{1}}{\left(1/\eta_{1}\right)-\eta_{2}+\left(\phi_{2}/2\eta_{2}\right)}, \end{array}$$

(14) $$\begin{array}{c} \eta_{\text{mix}}=\eta_{1}+\frac{\phi_{2}}{\left(1/\eta_{2}\right)-\eta_{1}+\left(\phi_{1}/2\eta_{1}\right)}, \end{array}$$

where “*η*_1_” is the viscosity of Isabgol husk dispersion, “*η*_2_” the viscosity of gum katira dispersion, “*ϕ*_1_” the volume fraction of Isabgol husk dispersion, and “*ϕ*_2_” is the volume fraction of gum katira dispersion, respectively.

### 2.4. Statistical Analysis

The results of different parameters studied were treated statistically and the data were reported as mean ± standard deviation (SD) for three successive determinations (*n* = 3). Statistical analysis was performed by Student's *t*-test, ANOVA, and *F* test at 95% confidence level, using a statistical package (SigmaStat v.2.07, Systat Software Inc., San Jose, California).

## 3. Results and Discussion

### 3.1. Determination of Viscosity and Shear Stress of Isabgol Husk and Gum Katira Blends

In order to understand the flow behaviour of the dispersions before and after blending, the dependence of flow properties like viscosity, shear stress, and shear rate was also investigated. The composition of blends of gum katira and Isabgol husk with their rheoparameters is shown in Table 1. The study was performed in triplicate (*n* = 3) and the data were represented as mean with standard deviation. As the flow behaviour of hydrophilic dispersion depends upon speed of rotation (shear rate), all these parameters were analyzed at a particular speed (60 rpm or 1 sec^−1^) by maintaining the recommended rate of torque value. It was observed that, on increasing the concentration of Isabgol husk in blends, the viscosity was increased while a reverse effect was observed with gum katira dispersions. The impact of Isabgol husk on enhancement of viscosity of blends was considered due to its more water retention capacity as the values of viscosity of Isabgol husk and gum katira dispersion in 1% w/v dispersion were 137.6 ± 1.3531 mPa and 53.3 ± 2.6147 mPa, respectively, at 298.15°K. In all the dispersions, the viscosity of the blends was decreased on increasing the temperature from 298.15°K to 333.15°K. The change in viscosity was slightly less on increasing temperature from 298.15°K to 313.15°K but these changes in viscosity were predicted more significant on increasing the temperature up to 333.15°K (*P* < 0.05). All the hydrocolloids comprising polysaccharides interact with water, reducing its diffusion and stabilizing its presence, and water is retained specifically through direct hydrogen bonding or the structure of these polymers contains water within extensive inter- and intramolecular voids. As the interactions between hydrocolloids and water depend on hydrogen bonding, the thermal conditions influence the water retention capacity. Hence, on higher temperature, the retained water may come out from blends and results in lower viscosity. Also, the hydrocolloids retain their extended structures and give rise to mixed entanglement. These entanglements in blends of Isabgol husk and gum katira at higher concentration of Isabgol husk may lead to enhancement of viscosity. Moreover, large and conformationally stiff blends at higher concentration of Isabgol husk may present essentially static surfaces encouraging extensive structure in surrounding water and holding it for prolonged time.

### 3.2. Effect of Acidic and Basic Media on Rheological Behaviour of Isabgol Husk and Gum Katira

**Figure 1:**
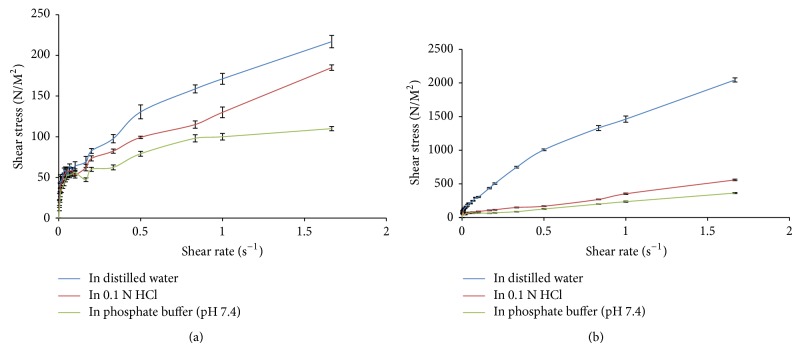
Flow behaviour of 1% w/v gum katira (a) and Isabgol husk (b) in distilled water, 0.1 N HCl, and phosphate buffer (pH 7.4) (the data used in the graph is the mean of three successive results (*n* = 3) and shown with error bars of SD).

Isabgol husk and gum katira being polysaccharide in nature are composed of different pentose and hexose branched chain structures with terminal hydrophilic groups and these are responsible for water retention as well as interaction with various ionic dispersing media. Moreover, the manufacturing conditions during their applicability in various processes may also have an impact on their flow characteristics. Hence, the effect of acidic (0.1 N HCl) and basic media (phosphate buffer pH 7.4) was analyzed on the flow pattern of 1% w/v aqueous dispersion of Isabgol husk and gum katira that are shown in Figure 1. The behaviour of husk and gum katira in 0.1 N HCl, phosphate buffer (pH 7.4) and water was pseudoplastic in nature (*n* < 1) but the pattern of shear stress at different shear rates in these media was statistically different (*P* < 0.05). The viscosity of Isabgol husk dispersion at 60 rpm in distilled water was higher than in 0.1 N HCl and phosphate buffer (pH 7.4) and the viscosities in these media were found to be 137.6 ± 1.3531, 76.2 ± 0.458, and 17.8 ± 0.488 mPa, respectively. The remarkable viscosity in distilled water may be due to water retention in branched chain structure of polysaccharides. The presence of acidic content in the form of galacturonic acid may interact with acidic media causing change in branched chain network and results in slow penetration of acidic dispersing media in polysaccharide structure. The phosphate buffer may have ionic effect and cause defragmentation of chains configuration of husk polysaccharides. The reported study has shown that, by enzymatic modification, the gelling hardness and adhesiveness of Isabgol husk can be reduced as high as 23% in convention enzymatic treatment and from 48% to 55% in solid-state enzymatic procedure, respectively [21–23]. The gel hardness and adhesiveness reduction of acid modified samples of Isabgol husk under reaction temperatures of 25°C and 37.5°C have also been found to be similar to solid-state enzymatic modification of Isabgol husk dispersion [4]. In the present study, the reduction in gel hardness and adhesiveness was thought to be due to HCl under 25°C and 37.5°C having comparable ability with enzymes such as Viscozyme L of breaking polysaccharide molecule networks. In the reported study, the sharp decrease in both hardness and adhesiveness of acid treated Isabgol husk at 50°C was analyzed due to the stronger reaction between HCl and Isabgol husk which altered the molecular structure of Isabgol husk and inhibited the formation of junction zones [24]. The rheological behaviour of 1% w/v gum katira was also different in acidic and basic conditions and the flow patterns were different from Isabgol husk in these media (*P* < 0.05).

### 3.3. Determination of Miscibility of Isabgol Husk and Gum Katira Blends

**Figure 2:**
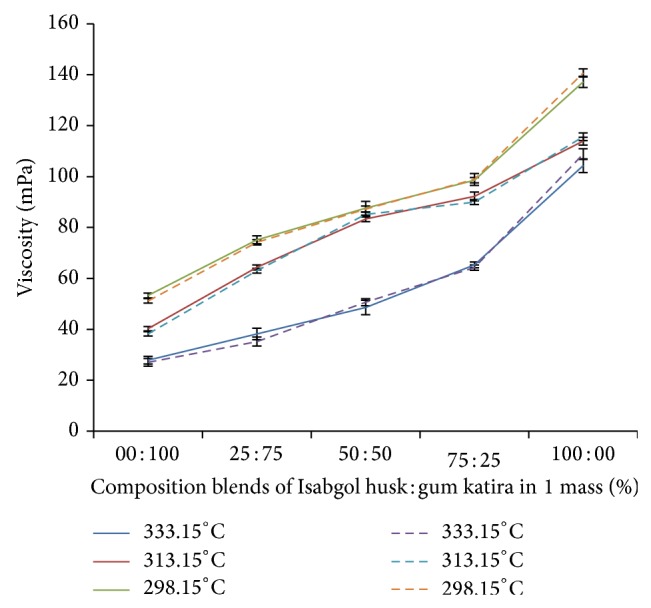
Comparative viscosity of blends determined experimentally (—) and by mathematical expressions (⋯) at 298.15°K, 313.15°K, and 333.15°K (the data used in the graph is the mean of three successive results (*n* = 3) and shown with error bars of SD).

According to Flory-Huggins [19], the free energy of mixing can be broken into two parts: an entropy part that always favors mixing and an enthalpy part that can either facilitate or prevent mixing, and it depends on the nature and intensity of the interaction between the two components. At a given temperature, complete, partial, or zero miscibility can be obtained with attractive (Flory-Huggins interaction parameter *χ* ≤ 0), weak repulsive (*χ* > 0), or strong repulsive (*χ* ≫ 0) interactions, respectively. Here, miscibility is the equilibrium composition of the two components above which the free energy of mixing is greater than zero, and phase separation is thermodynamically favorable. Generally, the immiscible blends of polymers show a negative deviation (Δ*b* < 0) as per (1) and (5) due to the heterogeneous nature of the components and results in phase separation, whereas positive deviation (Δ*b* > 0) is expected for the blends of comparatively higher solubility and homogeneous nature of the components [25]. The comparative analysis of experimental viscosity determination of different blends containing 1 mass% of Isabgol husk and 1 mass% of gum katira with mathematical analysis by (12), (13), and (14) has been shown in Figure 2. The viscosity determined by mathematical expressions and experimentations was similar and no significant difference (*P* > 0.05) was observed. The calculated values of “Δ*b*” for blends at 1 mass% of Isabgol husk and 1 mass% of gum katira with interaction parameter “*b*_12_” calculated theoretically by (3), (4), and (5) and experimentally are represented in Table 2. It was observed that the values of “Δ*b*” were increased on increasing the temperature from room temperature to 313.15°K and to 333.15°K, respectively, in all compositions of blends, and, at higher concentrations of Isabgol husk in blends (2 : 1), “Δ*b*” was found to be 42.64 at 298.15°K and 11.78 at 333.15°K, respectively. However, in blends comprising higher concentrations of gum katira (1 : 2), “Δ*b*” was 7.7 at 298.15°K and 3.69 at 333.15°K, respectively. These data of interaction parameter “Δ*b*” indicated the interaction and miscibility of blends at experimental thermal conditions and compositions of Isabgol husk and gum katira such as 1 : 1, 2 : 1, and 1 : 2 in mass% while more interaction and miscibility were depicted in blends containing comparatively higher Isabgol husk concentrations. The intrinsic viscosities of blends having polymer ratio 1 : 1 mass% at 298.15°K, 313.15°K, and 333.15°K were found to be 9.11 ± 1.0613, 20.786 ± 0.6932, and 29.885 ± 2.0232 mPa·s, respectively. The values of intrinsic viscosities of blends at 2 : 1 mass% (Isabgol husk : gum katira) were found to be 23.92 ± 1.210, 65.571 ± 2.6932, and 79.143 ± 1.8001 mPa·s at 298.15°K, 313.15°K, and 333.15°K, respectively. But, on increasing gum strength in blends as 2 mass%, the intrinsic viscosity was found lower than that obtained in higher strength of Isabgol husk. The results were 14 ± 2.0513, 27.179 ± 0.8562, and 49.286 ± 2.6320 mPa·s at 298.15°K, 313.15°K, and 333.15°K, respectively. The experimental data of intrinsic viscosities was similar to mathematical results obtained by (2) (*P* > 0.05).

### 3.4. Effect of Thermal Conditions on Consistency Index (*K*) and Flow Index (*n*) of Isabgol Husk-Gum Katira Blends

The flow behaviour of Isabgol husk-gum katira blends (1 : 1 mass%) indicated Newtonian flow pattern (e.g., viscosity was constant with increment of shear rate) at lower shear rate regions, while a shear-thinning flow behavior (e.g., viscosity decreased with increment of shear rate) was observed at higher shear rate regions. It seems that, at lower shear rates, there could be a constant formation and disruption of chain-chain entanglements and the rate of disruption with formation of the polymer chain entanglements could be at equilibrium; therefore, the viscosity might remain unchanged. As the shear rate increased, the rate of formation of entanglements could not keep up with the rate of disruption of the entanglements and resulted in decrement of viscosity with the increase in shear rate. The effect of shear rate was also observed on Isabgol husk dispersion and gum katira dispersion, but it was comparatively more and significant in blends as steep decrement in viscosity of dispersions was created on increment of shear stress.

The flow index (*n*) and consistency index (*K*) values obtained from the Power law model (9) for the blends at 298.15, 313.15, and 333.15°K are represented in Table 3. In all the blends, the value of “*n*” was deviated away from “1” (*n* < 1; *n* = 1 for Newtonian fluids) representing the shear-thinning flow behavior. The values of “*n*” obtained in all blends were decreased from 0.5132 ± 0.0091 to 0.2635 ± 0.0097 as the temperature was increased from 298.15°K to 333.15°K, respectively. But, the consistency index (*K*) was increased on increasing the temperature. The flow index (*n*) was decreased from 0.7070 ± 0.0086 to 0.2635 ± 0.0097 as the ratio of Isabgol in blends was changed from 100 to 25%, respectively, at 298.15°K. The decrement of flow index (*n*) on decreasing the Isabgol husk ratio in blends was observed at each temperature of the study and indicated the more profound effect of Isabgol husk on blends consistency. It may be due to a more pronounced shear-thinning flow behavior of the blends at lower husk concentration. This finding was consistent with Chun and Yoo [26], who reported that the “*n*” values for sweet potato flour dispersions were decreased while increasing the flour concentration. The viscoelastic nature of gum katira was lower than Isabgol husk as flow index (*n*) of gum katira dispersion was less than Isabgol husk. It has been reported that high temperature, shear, and pressure during extrusion usually lead to the degradation of macromolecular structure of polysaccharides, thereby resulting in a decrease in the molecular weight, and the polysaccharides with high molecular weight and rigid conformation exhibit a more distinct shear-thinning rheological behavior [27–29]. It has also been reported that a decrease in the molecular weight of polysaccharides can lead to the reduction of their viscosity [30]. The considerable changes in flow index (*n*), apparent viscosity (*η*_app_), and consistency index (*K*) were analyzed on changing the temperature and concentration of Isabgol husk and gum katira in blends that may be due to conformational change in structure of Isabgol husk and gum katira. On increasing husk strength from 25 to 100% in blends at 313.15°K, consistency index (*K*) was found to be 1.6163 ± 0.0063 and 1.9063 ± 0.0422 mPa·s, respectively, and, at 333.15°K, the increment in consistency index (*K*) was observed from 1.6922 ± 0.0145 to 2.2989 ± 0.2989 mPa·s, respectively. Similarly, the apparent viscosity was increased from 1.902 ± 1.6301 mPa·s to 20.303 ± 1.8905 mPa·s at 313.15°K and from 1.1170 ± 0.9652 mPa·s to 16.510 ± 1.0607 mPa·s at 333.15°K, respectively. The increment in the values of “*η*_(app)_” and “*K*” for the blends at the higher concentration of husk could be attributed to the greater number of junction zones as the polysaccharides are generally known to form junction zones in solution which prevents flow [31]. It is plausible in the present study that the molecules could be reached closer to one another and junction zones could be more readily formed while increasing the husk concentration, which could cause an increase in the “*η*_(app)_” and “*K*” values of blends at higher concentrations.

### 3.5. Effect of Temperature on Activation Energy (*E*_*act*_) of Isabgol Husk and Gum Katira Blends

The temperature dependence on apparent viscosity (*η*_app_) of polysaccharide dispersions was described by an Arrhenius model (11) [32]. In the present study, the values of *E*_act_ and constant *A* were determined for Isabgol husk, gum katira, and their blends from the correlation analysis of 1/*T* versus ln *η*_(app)_. The activation energy (*E*_act_) of blends in 1 mass% dispersion was determined in temperature range from 298.15°K to 333.15°K, respectively. The values of “*E*_act_” for Isabgol and gum katira dispersion were found to be 516.423 ± 2.0696 and 149.06 ± 0.9508 kJ/mol, respectively. The effect of Isabgol concentrations in blends was also remarkable on activation energy as on increasing the percentage ratio of Isabgol husk in blends in comparison to gum katira such as 25 : 75, 50 : 50, and 75 : 25, and the values of “*E*_act_” for blends were 88.906 ± 1.6052, 96.047 ± 0.8662, and 138.324 ± 1.0805 kJ/mol, respectively. The results obtained for “*E*_act_” were found with high correlation coefficients (0.99 < *r* ^2^), pointing out that the dependence of *η*_app 60 rpm_, for the blends, Isabgol husk, and gum katira, on experimental temperature followed the Arrhenius equation. According to Kim and Yoo [33], the trend of decreasing the viscosity at higher temperature can be associated with the increases in the intermolecular distances as a result of thermal expansion with increased temperature [33]. Furthermore, in the present study, on increasing the husk ratio in blends, the activation energy was increased and was considered due to formation of dense polymeric network that required more energy to flow and the movements of chains could have become more vigorous as the temperature was increased. This increment in temperature thereby resulted in the breakdown of some weakly associated interaction, that is, some smaller junction zones with lesser amounts of hydrogen bonds involved. The loss of this part of associations could lead to a decrease in the viscosity of blends, Isabgol husk, and gum katira dispersions.

### 3.6. Effect of Temperature and Determination of Plastic Viscosity and Yield Stress

Plastic or “Casson” fluids are fluidizing bodies characterized by a “yield stress” (or yield point) and with slowly decreasing viscosity at higher shear rates. Other liquid-like materials reach a constant viscosity but only after reaching their yield stress; these are called “Bingham fluids.” On increasing the shear rate, the viscosity is gradually decreased in Bingham, Casson, and Casson chocolate fluids; these are considered as shear-thinning systems. Structured fluids often do not flow unless they have reached a critical stress level called the “yield stress,” below which a material is “fully elastic” and above which the structure of the material breaks and it starts to flow. The plastic viscosity and yield stress obtained by Casson equation (6) are represented in Table 4. It was observed that yield stress was increased from 2.1277 ± 0.2700 to 2.3035 ± 0.0976 N/m^2^ at 298.15°K when the percentage ratio of Isabgol husk was increased from 25 to 75%, respectively, in blends. The plastic viscosity was also affected by increasing the ratio of Isabgol husk in blends as it was found to be 1.5662 ± 0.0036, 1.8007 ± 0.0162, and 2.4214 ± 0.0748 mPa·s in 25 : 75, 50 : 50, and 75 : 25 blends of Isabgol and gum katira, respectively, at 298.15°K. With an increase in gum content, the intermolecular gap in polysaccharides macromolecules of Isabgol husk may increase that may cause decrement in plastic viscosity on higher strength of gum in blends. A simultaneous increase in plastic viscosity and yield stress has been observed in Casson chocolate (7) and Bingham model (8) by increasing Isabgol husk content in blends as shown in Tables 5 and 6. The change in yield stress is different statistically than plastic viscosity (*P* < 0.05). This can be explained by the fact that, on adding higher concentration of Isabgol husk, the consistency of the blend could not be affected to a greater extent as both of the polysaccharides dispersions were of significant strength in themselves and it resulted in a slight change in plastic viscosity or flow of the blend. But the higher content of husk was intermixed with polymeric network of gum polysaccharide to a greater extent and the newly developed interlinked blend polymer network created a significant change in yield stress. It is well known that the yield value arises mainly from the interactions between the solid particles [34]. It was observed that the plastic viscosity and yield stress determined by all models were decreased in all blends on increasing the temperature from 298.15°K to 333.15°K. But a regular increment in plastic viscosity and yield stress was even analyzed on having higher concentration of Isabgol husk. It can be explained due to remarkable interlinking of gum and husk polysaccharide structure even at higher temperature. In IPC paste analysis, the consistency multiplier as shown in Table 7 was also changed on changing the husk strength and the pattern of decrement was similar to plastic viscosity and yield stress. The sequence of plastic viscosity data of the hydrodispersions at all thermal ranges was Isabgol husk > Isabgol husk-gum katira > gum katira. However, the plastic viscosity data and yield stress data were slightly higher for Bingham model than those obtained from Casson and Casson chocolate equation. The deviation of the fitted parameters for Bingham equation was <0.05% when compared to ~1% of deviation obtained for Casson and Casson chocolate equation.

## 4. Conclusion

The miscibility of Isabgol husk and gum katira blends in equal proportions as well as in higher concentrations of one another was found at studied thermal conditions. The blends and their components such as Isabgol husk and gum katira were found to be pseudoplastic in viscosity behaviour as, on increasing the shear stress, the viscosity was decreased down. The pseudoplastic behaviour was also revealed in acidic and basic media with remarkable results. The significant effect on rheological behaviour was shown by Isabgol husk as on higher concentration of husk; the plastic viscosity and yield stress were increased as analyzed by Bingham, Casson, and Casson chocolate model. The effect of temperature on viscosity was found according to Arrhenius equation, and in blends containing higher concentration of Isabgol husk than gum katira, more energy was required to start the flow. The results of miscibility of Isabgol husk and gum katira blends proved that the blends of these biopolymers may have potential in food and pharmaceutical industries. The pseudoplastic flow pattern in acidic and basic media may also play significant role in blends performance during food processing. In drug delivery systems, these blends may act as modulator for drug release in different environmental conditions of* in vitro* as well as* in vivo* studies. Also, applicability of the shear-thinning/pseudoplastic behaviour of these blends may also be advantageous for easy flow of lava, ketchup, jellies, gems, nail polish, paint, cream, paste, ointment, and varnish and even for some polymeric solutions from the containers.

## Figures and Tables

**Table 1:** Viscosity and shear stress of Isabgol husk, gum katira, and their blends having 1 : 1 mass% of Isabgol husk and gum katira on 298.15°K, 313.15°K, and 333.15°K at 60 rpm (1 sec.^−1^).

Composition of Isabgol husk and gum katira	Viscosity (mPa) (mean ± SD)∗	Shear stress (N/m^2^) (mean ± SD)
At 298.15°K		
100 : 0	137.6 ± 1.3531	1496.75 ± 1.6833
75 : 25	98.5 ± 1.5527	1301.85 ± 4.2933
50 : 50	88.6 ± 2.4682	1089.23 ± 3.9310
25 : 75	75.5 ± 3.6669	720.55 ± 2.6253
0 : 100	53.3 ± 2.6147	510.26 ± 4.5279
At 313.15°K		
100 : 0	114.3 ± 3.5369	1244.58 ± 3.8743
75 : 25	92.9 ± 1.3917	1001.85 ± 4.6793
50 : 50	83.7 ± 1.2326	951.54 ± 5.6126
25 : 75	64.5 ± 3.1119	669.62 ± 5.8796
0 : 100	40.1 ± 1.5991	439.02 ± 3.1245
At 333.15°K		
100 : 0	106 ± 2.7203	908.65 ± 2.8533
75 : 25	63.5 ± 3.4664	654.55 ± 5.4932
50 : 50	48.8 ± 1.9902	558.73 ± 4.6455
25 : 75	29.3 ± 0.6741	338.95 ± 3.0102
0 : 100	27.2 ± 2.1417	209.04 ± 3.1193

∗Average of three successive results (*n* = 3), S.D is the standard deviation.

**Table 2:** Miscibility parameters of Isabgol husk and gum katira blends at 298.15°K, 313.15°K, and 333.15°K.

Mass% composition of Isabgol-gum katira	At 298.15°K		Δ*b*	At 313.15°K		Δ*b*	At 333.15°K		Δ*b*
	*b*12^*^, experimental	*b*12, theoretical		*b*12, experimental	*b*12, theoretical		*b*12, experimental	*b*12, theoretical	
1 : 0	—	—	—	—	—	—	—	—	—
1 : 1	8.10 ± 0.1350	6.3 ± 0.0305	1.8	32.41 ± 0.6368	14.82 ± 0.5107	17.59	28.56 ± 0.4201	19.43 ± 0.4968	9.13
2 : 1	48.94 ± 1.1302	6.3 ± 0.0305	42.64	39.00 ± 0.5430	14.82 ± 0.5107	24.18	31.21 ± 0.6179	19.43 ± 0.4968	11.78
1 : 2	14.00 ± 0.2709	6.3 ± 0.0305	7.7	26.65 ± 0.3873	14.82 ± 0.5107	11.83	23.12 ± 0.3513	19.43 ± 0.4968	3.69
1 : 0	—	—	—	—	—	—	—	—	—

∗Average of three successive results (*n* = 3) and “±” value is the standard deviation.

**Table 3:** Consistency index (*K*) and flow index (*n*) of blends in 1 : 1 mass% of Isabgol husk and gum katira at 298.15°K, 313.15°K, and 333.15°K.

%Composition (Isabgol husk : gum katira)	298.15°K		313.15°K		333.15°K	
	Consistency index (*K*) (mPa·s)∗	Flow index (*n*)	Consistency index (*K*) (mPa·s)	Flow index (*n*)	Consistency index (*K*) (mPa·s)	Flow index (*n*)
100 : 00	1.9025 ± 0.0092	0.7070 ± 0.0086	1.9063 ± 0.0422	0.6387 ± 0.0094	2.2989 ± 0.0387	0.5818 ± 0.011
75 : 25	1.6790 ± 0.0510	0.5132 ± 0.0091	1.7098 ± 0.0342	0.3833 ± 0.0119	1.8147 ± 0.1225	0.2747 ± 0.0074
50 : 50	1.6846 ± 0.0510	0.3893 ± 0.0111	1.6582 ± 0.0858	0.2771 ± 0.0145	1.7721 ± 0.0387	0.2145 ± 0.0120
25 : 75	1.5705 ± 0.0469	0.2635 ± 0.0097	1.6163 ± 0.0063	0.1548 ± 0.0079	1.6922 ± 0.0145	0.1271 ± 0.0077
00 : 100	1.2732 ± 0.0044	0.6323 ± 0.0117	1.8864 ± 0.0489	0.5195 ± 0.0065	1.9665 ± 0.0161	0.4995 ± 0.0059

∗Average of three successive results (*n* = 3) and “±” value is the standard deviation.

**Table 4:** Plastic viscosity and yield stress data of 1 : 1 mass% blends calculated from Casson equation.

%Composition (Isabgol husk : gum katira)	298.15°K		313.15°K		333.15°K	
	Plastic viscosity (mPa·s)∗	Yield stress (N/m^2^)	Plastic viscosity (mPa·s)	Yield stress (N/m^2^)	Plastic viscosity (mPa·s)	Yield stress (N/m^2^)
100 : 00	3.9661 ± 0.0524	2.464853 ± 0.0529	4.1064 ± 0.0098	2.4715 ± 0.0220	3.8795 ± 0.0507	2.1034 ± 0.1813
75 : 25	2.4214 ± 0.0748	2.3035 ± 0.0976	1.8224 ± 0.0494	2.2210 ± 0.0314	1.4252 ± 0.0136	1.7308 ± 0.0229
50 : 50	1.8007 ± 0.0162	2.2164 ± 0.0090	1.1622 ± 0.0084	2.0283 ± 0.1088	1.0341 ± 0.0481	1.7891 ± 0.0464
25 : 75	1.5662 ± 0.0036	2.1277 ± 0.270	1.3312 ± 0.0225	1.8086 ± 0.0370	1.1841 ± 0.0671	0.7002 ± 0.0119
00 : 100	1.2016 ± 0.0114	1.4243 ± 0.0345	1.1368 ± 0.0094	1.1979 ± 0.0084	1.8883 ± 0.0120	2.1008 ± 0.0495

∗Average of three successive results (*n* = 3) and “±” value is the standard deviation.

**Table 5:** Plastic viscosity and yield stress data of 1 : 1 mass% blends calculated from Casson chocolate equation.

%Composition (Isabgol husk : gum katira)	298.15°K		313.15°K		333.15°K	
	Plastic viscosity (mPa·s)∗	Yield stress (N/m^2^)	Plastic viscosity (mPa·s)	Yield stress (N/m^2^)	Plastic viscosity (mPa·s)	Yield stress (N/m^2^)
100 : 00	3.9692 ± 0.0459	59.083 ± 0.5798	3.8682 ± 0.0579	57.938 ± 0.7718	3.4452 ± 0.1557	50.464 ± 0.6303
75 : 25	2.7114 ± 0.0557	54.891 ± 1.1219	2.4224 ± 0.0557	49.093 ± 0.6980	1.3252 ± 0.0645	42.635 ± 1.1956
50 : 50	1.8486 ± 0.0272	48.277 ± 0.9902	1.4622 ± 0.0272	37.286 ± 0.7415	1.1872 ± 0.0114	33.214 ± 0.7813
25 : 75	1.2497 ± 0.0224	38.023 ± 0.3059	1.1312 ± 0.0224	32.292 ± 0.8600	1.0841 ± 0.0325	31.022 ± 0.6193
00 : 100	1.5016 ± 0.0112	19.762 ± 0.3071	1.1368 ± 0.0112	21.609 ± 0.9596	1.1183 ± 0.0889	13.963 ± 0.7606

∗Average of three successive results (*n* = 3) and “±” value is the standard deviation.

**Table 6:** Plastic viscosity and yield stress data of 1 : 1 mass% blends from Bingham equation.

%Blend composition (Isabgol husk : gum katira)	298.15°K		313.15°K		333.15°K	
	Plastic viscosity (mPa·s)∗	Yield stress (N/m^2^)	Plastic viscosity (mPa·s)	Yield stress (N/m^2^)	Plastic viscosity (mPa·s)	Yield stress (N/m^2^)
100 : 00	4.9644 ± 0.0566	54.249 ± 0.7823	4.1303 ± 0.0252	49.61 ± 0.3671	3.6151 ± 0.07104	51.42 ± 0.6080
75 : 25	3.9867 ± 0.0812	47.991 ± 0.5249	3.5824 ± 0.1096	46.379 ± 0.6249	3.3013 ± 0.0605	40.33 ± 0.8000
50 : 50	3.7473 ± 0.1575	44.381 ± 0.7967	2.9842 ± 0.0718	33.03 ± 0.8479	1.8621 ± 0.0163	30.21 ± 0.5445
25 : 75	2.8009 ± 0.1065	36.024 ± 1.0205	2.2002 ± 0.0793	27.39 ± 0.5631	1.9173 ± 0.0634	25.06 ± 0.7350
00 : 100	2.8747 ± 0.0688	25.064 ± 1.030	2.6609 ± 0.1024	23.62 ± 0.5337	1.9988 ± 0.0886	18.78 ± 0.8113

∗Average of three successive results (*n* = 3) and “±” value is the standard deviation.

**Table 7:** Consistency multiplier data of 1 : 1 mass% blends calculated from IPC paste analysis.

%Blend composition (Isabgol husk : gum katira)	298.15°K	313.15°K	333.15°K
	Consistency multiplier∗	Consistency multiplier	Consistency multiplier
100 : 00	6.2235 ± 0.0256	5.7790 ± 0.1023	2.9622 ± 0.0287
75 : 25	5.0672 ± 0.0869	4.6648 ± 0.1161	3.4512 ± 0.0511
50 : 50	4.5448 ± 0.1164	3.8566 ± 0.1255	2.5612 ± 0.0835
25 : 75	4.0724 ± 0.0828	2.0024 ± 0.0597	2.0754 ± 0.0827
00 : 00	4.4421 ± 0.0734	4.5035 ± 0.0071	3.6218 ± 0.0116

∗Average of three successive results (*n* = 3) and “±” value is the standard deviation.
